# Atypical and Polymorphic Presentation of Lichen Planus in a 16-Year-Old Boy: A Rare and Intriguing Case

**DOI:** 10.7759/cureus.112624

**Published:** 2026-07-13

**Authors:** Amrita Shruti, Hemant Singh, Shiv S Jha, Ratan Kumar

**Affiliations:** 1 Dermatology, Netaji Subhas Medical College and Hospital, Jamshedpur, IND; 2 Dermatology, All India Institute of Medical Sciences, Gorakhpur, Gorakhpur, IND; 3 Pediatrics and Neonatology, Netaji Subhas Medical College and Hospital, Jamshedpur, IND; 4 Pediatrics, Manipal Tata Medical College, Manipal Academy of Higher Education (MAHE), Jamshedpur, IND; 5 Pediatrics, Tata Main Hospital, Jamshedpur, IND

**Keywords:** adolescent, keratolytic treatment, lichen planus, polymorphic, porokeratosis

## Abstract

Lichen planus (LP) is an inflammatory condition of the skin characterized clinically by purple, polygonal, pruritic papules and histopathologically by lymphocytic infiltrate at the dermoepidermal junction. Atypical hypertrophic, atrophic, and annular forms have also been described in the literature. We report a rare case of polymorphic LP in a 16-year-old male with a 10-year history of mildly pruritic, hyperpigmented lesions involving the trunk, extremities, genitalia, and scrotum. Clinical examination revealed a mix of classical LP papules, verrucous/rupoid plaques, annular porokeratosis-like lesions, and areas of erosion. Dermoscopy showed Wickham’s striae; histopathology demonstrated a lichenoid lymphocytic infiltrate, saw-tooth acanthosis, wedge-shaped hypergranulosis, colloid bodies, and compact orthohyperkeratosis, confirming LP. The patient responded well to a short systemic steroid taper combined with a topical potent corticosteroid (mometasone) and keratolytic therapy. This case highlights the diagnostic challenge of polymorphic LP in adolescents and underscores the value of dermoscopy and histology in atypical presentations.

## Introduction

Lichen planus (LP) is an idiopathic inflammatory disease affecting the skin, hair, nails, and mucous membranes. Classical cutaneous LP is characterized clinically by the “four Ps”: purple, polygonal, pruritic papules; histologically, it shows a dense, band-like lymphocytic infiltrate at the dermoepidermal junction with acanthosis, wedge-shaped hypergranulosis, basal cell layer destruction, and apoptotic keratinocytes (colloid or Civatte bodies) [1]. Cutaneous LP affects approximately 0.2-1% of adults, while oral lesions are seen in up to 1-4% of the population. The disease most commonly begins in the fifth or sixth decade; two-thirds of patients develop LP between 30 and 60 years of age, and only 1-4% of patients are pediatric. However, recent studies suggest LP may be under-recognized in children. Although some series report female predominance (up to 2:1), overall gender predilection is variable [2].

Beyond classical papules and plaques, LP has multiple morphological and distributional variants, including annular, linear, atrophic, hypertrophic, inverse, eruptive, bullous, ulcerative, pigmentosus, lichen planopilaris, vulvovaginal, actinic, overlap syndromes, and LP pemphigoides. Rare atypical forms, including psoriasiform, retiform, porokeratosis-like, and pyoderma gangrenosum-like, have also been described [3-6]. We report the case of a 16-year-old boy with a 10-year history of polymorphic LP combining classical and several atypical morphologies, which posed diagnostic and therapeutic challenges.

## Case presentation

A 16-year-old boy presented with multiple hyperpigmented skin lesions of 10 years’ duration. Lesions were mildly pruritic and involved the neck, chest, back, upper and lower limbs, penis, and scrotum. Hair, nails, and oral mucosa were spared.

**Figure 1:**
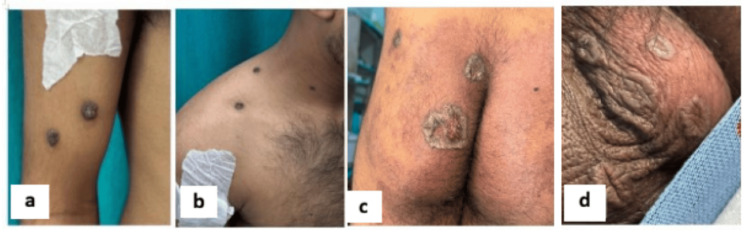
Skin lesions over the (a) arm, (b) shoulder, (c) buttocks, and (d) scrotum.

Examination showed multiple well-defined, discrete hyperpigmented verrucous plaques measuring 2-4 cm with adherent crusts and erythematous-to-hyperpigmented borders over the neck, axillae, upper limbs, chest, and back. Lesions over the buttocks, penis, scrotum, and feet were annular plaques with verrucous surfaces in some areas and smooth surfaces in others. Some lesions were nodular; the largest on the buttock measured approximately 5 cm. Several lesions were cauliflower-like, resembling rupoid psoriasis, with adherent crusts and scales. Lesions on the feet, legs, and scrotum had a prominent thready hyperkeratotic margin and were positive on the “thread” (gentian violet ink) test, resembling porokeratosis. A few lesions showed central erosion (Figure 1). Only a minority displayed classical LP morphology.

**Figure 2:**
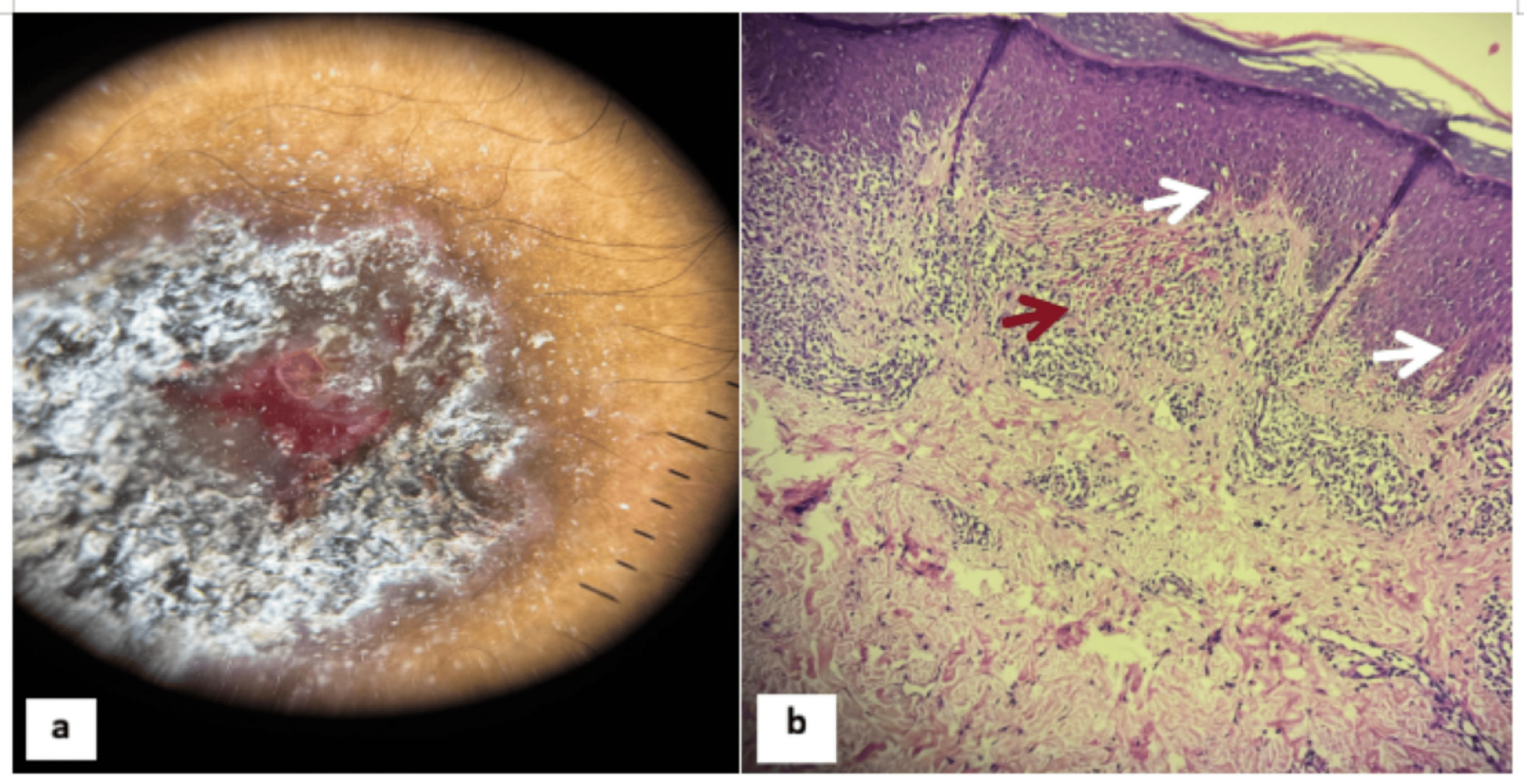
(a) Dermoscopy showing Wickham’s striae. (b) Histopathology image. The white arrows show focal hypergranulosis and acanthosis along with vacuolar degeneration of the basal layer of the skin. The red arrow shows a lymphocytic infiltrate with scattered apoptotic keratinocytes.

Dermoscopy revealed Wickham’s striae in most lesions, which are white crossing lines in a reticulated pattern (Figure 2a). Histopathology demonstrated a moderately dense superficial, band-like (lichenoid) infiltrate of lymphocytes, saw-tooth acanthosis, squamatization of the basal layer, focal wedge-shaped hypergranulosis, scattered necrotic keratinocytes (colloid bodies), and mild compact orthohyperkeratosis, features consistent with LP (Figure 2b).

The patient received systemic steroids in the form of oral prednisolone 1 mg/kg/day, tapered after one week; oral levocetirizine 5 mg at bedtime; topical mometasone with 3.5% salicylic acid applied twice daily to thick lesions; and plain mometasone cream twice daily to thin lesions. He responded well initially, but was later lost to follow-up.

## Discussion

This case is notable for the patient’s age, long disease duration, and striking polymorphism. LP typically affects adults and often follows a self-limited course of one to two years; pediatric cases and prolonged disease courses are uncommon. Most published atypical LP reports describe monomorphic presentations mimicking a single disease (e.g., psoriasis-like or porokeratosis-like LP).

Papagiorgiou et al. reported a case of LP presenting clinically as psoriasis in a 60-year-old female [3]. Their case had all the lesions resembling chronic plaque-type psoriasis, and the final diagnosis of LP was made with the help of dermoscopy and histopathological study. In contrast, our case had few lesions resembling rupoid psoriasis, and dermoscopy and histopathological investigation helped confirm the diagnosis in our case as well. Singh et al. reported a case of LP presenting as porokeratosis in a 44-year-old woman for the past two years [4]. Sharma reported a case of LP in a 64-year-old lady presenting as cellulitis, which responded not with antibiotics but with systemic steroids [5]. Mirchandani et al. reported a case of generalized hypertrophic LP in a 58-year-old female with extremely verrucous and exophytic lesions not usually seen in hypertrophic LP [6]. Their case also had a monomorphic presentation. In almost all cases, the patient was a middle-aged female, and the disease duration was less than two years. Thus, our case being that of a 16-year-old boy with a duration of over 10 years and a varied polymorphic and atypical presentation of LP (coexisting classical LP, rupoid psoriasis-like lesions, erosive areas, and porokeratosis-like plaques) posed a challenge not only in diagnosis but also sheds new light on the pathophysiology of the disease, all the while demonstrating the importance of being familiar with rarer forms of LP.

Dermoscopy and histopathology were pivotal in reaching a unifying diagnosis. The presence of Wickham’s striae and classic lichenoid histology supported LP despite unusual clinical morphologies. This case underscores the importance of considering LP in the differential diagnosis of chronic, hyperkeratotic, verrucous, or annular lesions in adolescents, and demonstrates that LP can manifest in polymorphic forms over prolonged periods.

Corticosteroids (topical, systemic, and intralesional) are considered first-line therapy for all forms of LP. For severe forms and cases not responding to steroids, other treatment options include topical calcineurin Inhibitors, systemic immunosuppressants and retinoids, and psoralen and ultraviolet rays [7,8].

## Conclusions

This case report highlights the unusual presentation of polymorphic LP in a pediatric patient with a remarkably prolonged disease duration. The coexistence of classical LP morphology with multiple atypical variants represents a unique presentation of the disease. Recognition of atypical and mixed morphological presentations, corroborated by dermoscopy and histopathology, is essential to avoid misdiagnosis and guide appropriate therapy.
